# Pleiotropic effects of Syntaxin16 identified by gene editing in cultured adipocytes

**DOI:** 10.3389/fcell.2022.1033501

**Published:** 2022-11-18

**Authors:** Shaun K. Bremner, Woroud S. Al Shammari, Roderick S. Milligan, Brian D. Hudson, Calum Sutherland, Nia J. Bryant, Gwyn W. Gould

**Affiliations:** ^1^ Strathclyde Institute of Pharmacy and Biomedical Sciences, University of Strathclyde, Glasgow, United Kingdom; ^2^ The Centre for Translational Pharmacology, Institute of Molecular, Cell and Systems Biology, University of Glasgow, Glasgow, United Kingdom; ^3^ Department of Cellular Medicine, Ninewells Hospital, University of Dundee, Glasgow, United Kingdom; ^4^ Department of Biology, University of York, York, United Kingdom

**Keywords:** syntaxin 16 (STX16), GLUT4, adipocyte, adipokine, CRISPR

## Abstract

Adipocytes play multiple roles in the regulation of glucose metabolism which rely on the regulation of membrane traffic. These include secretion of adipokines and serving as an energy store. Central to their energy storing function is the ability to increase glucose uptake in response to insulin, mediated through translocation of the facilitative glucose transporter GLUT4 to the cell surface. The trans-Golgi reticulum localized SNARE protein syntaxin 16 (Sx16) has been identified as a key component of the secretory pathway required for insulin-regulated trafficking of GLUT4. We used CRISPR/Cas9 technology to generate 3T3-L1 adipocytes lacking Sx16 to understand the role of the secretory pathway on adipocyte function. GLUT4 mRNA and protein levels were reduced in Sx16 knockout adipocytes and insulin stimulated GLUT4 translocation to the cell surface was reduced. Strikingly, neither basal nor insulin-stimulated glucose transport were affected. By contrast, GLUT1 levels were upregulated in Sx16 knockout cells. Levels of sortilin and insulin regulated aminopeptidase were also increased in Sx16 knockout adipocytes which may indicate an upregulation of an alternative GLUT4 sorting pathway as a compensatory mechanism for the loss of Sx16. In response to chronic insulin stimulation, Sx16 knockout adipocytes exhibit elevated insulin-independent glucose transport and significant alterations in lactate metabolism. We further show that the adipokine secretory pathways are impaired in Sx16 knockout cells. Together this demonstrates a role for Sx16 in the control of glucose transport, the response to elevated insulin, cellular metabolic profiles and adipocytokine secretion.

## 1 Introduction

Regulation of blood glucose is orchestrated by the hormones insulin and glucagon. Postprandially, insulin stimulates striated muscles and adipocytes to increase glucose uptake. This is principally achieved through the rapid mobilization of glucose transporter 4 (GLUT4) to the cell surface. In the absence of insulin, GLUT4 is sequestered intracellularly in vesicles designated the GLUT4 storage compartment (GSC). A subset of these vesicles, termed insulin responsive vesicles (IRVs), are the downstream effectors of insulin signaling (Gould et al., 2020). Upon insulin stimulation, IRVs translocate to the cell surface enabling glucose uptake into skeletal muscles and adipocytes.

Insulin resistance in obesity and type 2 diabetes mellitus (T2DM) is characterized by decreased insulin-stimulated glucose transport and metabolism in adipocytes and skeletal muscle. GLUT4 is down regulated in adipocytes of patients with T2DM (W. T. Garvey et al., 1992), and this in turn is associated with systemic insulin resistance (Garvey et al., 1988; Abel et al., 2001; Kahn, 2018; Santoro et al., 2021). Both GLUT4 and the insulin-regulated aminopeptidase (IRAP) (a protein which displays a similar insulin-stimulated translocation to the plasma membrane as GLUT4) are redistributed to higher density intracellular membrane fractions in T2DM and do not translocate to the cell surface in response to insulin (Garvey et al., 1998; Maianu et al., 2001). As a consequence, there has been intense research on how GLUT4 is sorted into IRVs.

Previous studies have implicated the Q_a_-SNARE syntaxin 16 (Sx16) in the control of GLUT4 trafficking (Perera et al., 2003; Shewan et al., 2003; Proctor et al., 2006; Roccisana et al., 2013). Sx16 co-localizes with GLUT4 and undergoes insulin-stimulated translocation to the plasma membrane (Shewan et al., 2003). Expression of a truncated form of Sx16 lacking its transmembrane domain, which acts in a dominant negative manner, delays the reversal of insulin-stimulated glucose transport, suggesting a role for Sx16 in sorting GLUT4 from endosomes into GSC (Proctor et al., 2006). Consistent with this, transient knockdown of Sx16 expression in 3T3-L1 adipocytes reduces insulin-stimulated GLUT4 translocation to the plasma membrane and glucose uptake (Proctor et al., 2006). While informative, these studies did not consider the wider role of Sx16 in adipocyte biology and measurements of, for example, glucose transport, are hampered by population effects in which some cells exhibit high levels of knockdown, others less so (Wollman et al., 2022).

In this investigation CRISPR/Cas9 was utilized to generate a Sx16 knockout 3T3-L1 cell line so as to facilitate a wider understanding of the role of Sx16 in adipocyte biology. A 2 base pair mutation was introduced into the Sx16 gene resulting in Sx16 knockout by premature truncation of the protein at codon 15. Sx16 knockout adipocytes had reduced levels of GLUT4 and insulin-stimulated GLUT4 translocation, but insulin-stimulated glucose uptake was not affected. We also observed that Sx16 knockout cells exhibited defective adiponectin secretion and reduced lipopolysaccharide (LPS)-induced monocyte chemoattractant protein-1 (MCP-1) secretion, suggesting that Sx16 plays pleiotropic effects on adipocyte function. Given the central role of adipocytes in metabolic regulation, we considered whether Sx16 knockout cells exhibit distinct responses to hyperinsulinemia, and observed marked changes in glucose transport and lactate metabolism compared to wild type cells which may have important implications for the development of metabolic disease.

## 2 Materials and methods

### 2.1 3T3-L1 cell culture

3T3-L1 fibroblasts were obtained from the American Tissue Culture Collection (#CL-173). Fibroblasts were maintained in Dulbecco’s modified Eagle’s Medium (DMEM) supplemented with 10% (v/v) new-born calf serum and 100 U/ml (v/v) penicillin and streptomycin. Cells were cultured at 37°C in a humidified atmosphere of 10% (v/v) CO_2_. Differentiation into adipocytes was carried out exactly as outlined in (Katwan et al., 2019). Cells were used 10 or 11 days after induction of adipogenesis.

### 2.2 CRISPR genome editing

3T3-L1 fibroblasts were seeded in a 12-well plate 24 h prior to experimentation. gRNA was purchased from Merck (Gillingham, United Kingdom) with the sequence 5′-CCG​CAA​CAA​CAA​GAA​AGC​GU which recognized the N-terminus of Sx16. Ribonucleoprotein (RNP) particles were generated by combining 600 ng gRNA and 2 µg Cas9-GFP (Merck, Gillingham, United Kingdom). RNP were transfected into fibroblasts using Xfect™ Transfection Reagent (TaKaRa bio, Göteborg, Sweden) according to the manufacturer’s instructions. The following day, media was replaced, and cells incubated for 48 h. Following this, cells were seeded into 96-well plates and single cell colonies expanded. Genomic DNA was extracted using QuickExtract™ DNA extraction Solution (Cambio LTD., Cambridge, United Kingdom). For sequencing, primers were designed around the gRNA target sequence; Forward 5′-GAG​TGG​AAT​CAG​CTA​GGC and reverse 5′-ACA​CGG​TGT​GTG​TGT​CTG​GA. DNA sequencing was performed by DNA Sequencing & Services (MRC I PPU, School of Life Sciences, University of Dundee, Scotland, www.dnaseq.co.uk) using Applied Biosystems Big-Dye Ver 3.1 chemistry on an Applied Biosystems model 3730 automated capillary DNA sequencer. Passage matched cells were transfected with Cas9-GFP alone to be used as a “wild type” control.

### 2.3 2-Deoxyglucose transport

2-deoxyglucose uptake was assayed as outlined in (Proctor et al., 2006). Adipocytes were incubated in serum free DMEM for 2 h followed by Krebs-Ringer phosphate (KRP) (128 mM NaCl, 4.7 mM KCl, 5 mM NaH_2_PO_4_, 1.25 mM MgSO_4_, 1.25 mM CaCl_2_) for 20 min prior to stimulation with insulin. Assays were initiated by the addition of 50 µM 2-deoxy-d-glucose and 0.5 µCi 2-[^3^H]-deoxy-D-glucose. Cells were incubated for 3 min followed by rapidly washing in ice-cold PBS. Adipocytes were lysed with 1% (v/v) triton X-100 and radioactivity determined by liquid scintillation counting. In parallel, cells were treated with 10 µM cytochalasin B to determine non-specific association of 2-[^3^H]-deoxyglucose (Bloch, 1973).

### 2.4 Oil Red O staining

Staining was carried out as described in (Katwan et al., 2019). Cells were fixed in 10% (v/v) formalin and washed briefly with 60% (v/v) isopropanol. Cells were stained with 5.14 mM Oil Red O in 60% (v/v) isopropanol for 10 min followed by 4 washes with water. Coverslips were dipped in Mayers Hematoxylin (Merck, Gillingham, United Kingdom) for 30 s followed by four washes in water prior to being mounted on glass microscope slides and photographed. For the quantification of Oil Red O stain, cells were incubated in 100% isopropanol for 15 min, the supernatant collected, and absorbance measured at 590 nm.

### 2.5 Cell lysate preparation and subcellular fractionation

Cells were scraped into lysis buffer (50 mM Tris-HCl, pH 7.4 at 4°C, 50 mM NaF, 1 mM Na_4_P_2_O_7_, 1 mM EDTA, 1 mM EGTA, 1% (v/v) Triton X-100, 250 mM mannitol, 1 mM DTT, Pierce™ Protease Inhibitor Tablet (Fisher Scientific, Loughborough, United Kingdom) and Phosphatase Inhibitor Cocktail Set II (Merck, Gillingham, United Kingdom)). Lysates were incubated on ice for 20 min before sedimentation at 21,910 x *g* for 5 min at 4°C. The supernatant was collected and stored at −20°C.

Subcellular fractionations were based on (Gould et al., 1989; Roccisana et al., 2013; Black et al., 2022). Adipocytes were homogenized in HEPES-EDTA-sucrose (HES) buffer (20 mM HEPES, 1 mM EDTA, 225 mM sucrose, pH 7.4) followed by successive centrifugation.

### 2.6 SDS-PAGE and immunoblotting

SDS-PAGE and immunoblotting was carried out as outlined by (Mancini et al., 2018; Black et al., 2022). Secondary antibody fluorescence was detected using the LI-COR Odyssey^®^ SA system. Band intensity was quantified with Image Studio lite software. Total protein was stained with Revert™ Total Protein Stain (Fisher Scientific, Loughborough, United Kingdom). Primary antibodies used are as follows; anti-adiponectin (Cell Signaling Technology Cat#2789), RRID:AB_2221630), anti-AKT (pan) (Cell Signaling Technology Cat# 2920, RRID:AB_1147620), anti-AKT Phospho-S473 (Cell Signaling Technology Cat#4058), RRID:AB_331168), anti-FAS (Cell Signaling Technology Cat#3180), RRID:AB_2100796), anti-IRAP (A generous gift from Susanna Keller, University of Virginia, United States), anti-MCT1 (Thermo Fisher Scientific Cat# MA5-18288, RRID:AB_2539662), anti-sortilin (Abcam Cat# ab16640, RRID:AB_2192606), anti-Sx6 (Abcam Cat# ab12370, RRID:AB_2196497), anti-Sx16 (Abcam Cat# ab134945) and anti-VAMP4 (Synaptic Systems Cat# 136,002, RRID:AB_887816). Anti-GLUT1 (Abcam Cat#ab115730, RRID:AB_10903230), anti-GLUT4 (A combination of rabbit polyclonal antibodies raised against the C terminus of GLUT4 and the N-terminus of GLUT4 (Brant et al., 1992) identified several species in the molecular weight range of 45–55 kDa, consistent with the reported heterogeneous glycosylation of these proteins. Quantification was performed on the entire region from 45–55 kDa as previously employed (Roccisana et al., 2013; Sadler et al., 2016).

### 2.7 RNA extraction and qPCR analysis

Total cellular RNA was extracted from 3T3-L1 cells using a Total RNA Miniprep Kit (Monarch^®^, T2010) according to the manufacturer’s instructions. Residual genomic DNA was removed using TURBO DNA-free™ Kit (Invitrogen, AM 1907) according to the manufacturer’s instructions. Reverse transcription reaction was carried out using M-MLV Reverse Transcriptase (Invitrogen, 28,025,013) according to the manufacturer’s instructions. Quantitative PCR (qPCR) was carried out using Fast SYBR^™^ Green Master Mix (Invitrogen, 4385612) and the primers outlined in Table 1. Reactions were incubated in a thermocycler at the following settings; (1) 50°C 2 min, (2) 95°C 2 min, (3) 95°C 15 s, (4) 58°C 1 min, then 44 cycles of stage 3 and 4, (5) 60°C 5 s, (6) 95°C 20 s.

**TABLE 1:** Sequences of the primers used in Real-Time PCR analysis.

Gene name	5′-3′	3′-5′
GLUT1	TCAACACGGCCTTCACTG	CAC​GAT​GCT​CAG​ATA​GGA​CAT​C
GLUT4	GTA​ACT​TCA​TTG​TCG​GCA​TGG	AGC​TGA​GAT​CTG​GTC​AAA​CG
Hypoxanthine-guanine phosphoribosyl-transferase	AGG​CCA​GAC​TTT​GTT​GGA​TTT​GAA	CAA​CTT​GCG​CTC​ATC​TTA​GGC​TTT

### 2.8 Other assays

MCP-1 secretion quantification was carried out using a mouse adiponectin Quantikine ELISA kit (R&D systems, Abingdon, United Kingdom) according to the manufacturer’s protocol. Lactate quantification was carried out using Lactate-Glo^™^ Assay (Promega, Southampton, United Kingdom) according to the manufacturer’s protocol.

### 2.9 Statistical analysis

Result are expressed as ± SD with the N-number indicated. Significant differences were determined using either Students *t*-test or two-way ANOVA, with *p* < 0.05 being deemed as significant. Statistical analysis was carried out using GraphPad Prism software.

## 3 Results

### 3.1 Generation and characterization of Sx16 knockout cells

**FIGURE 1:**
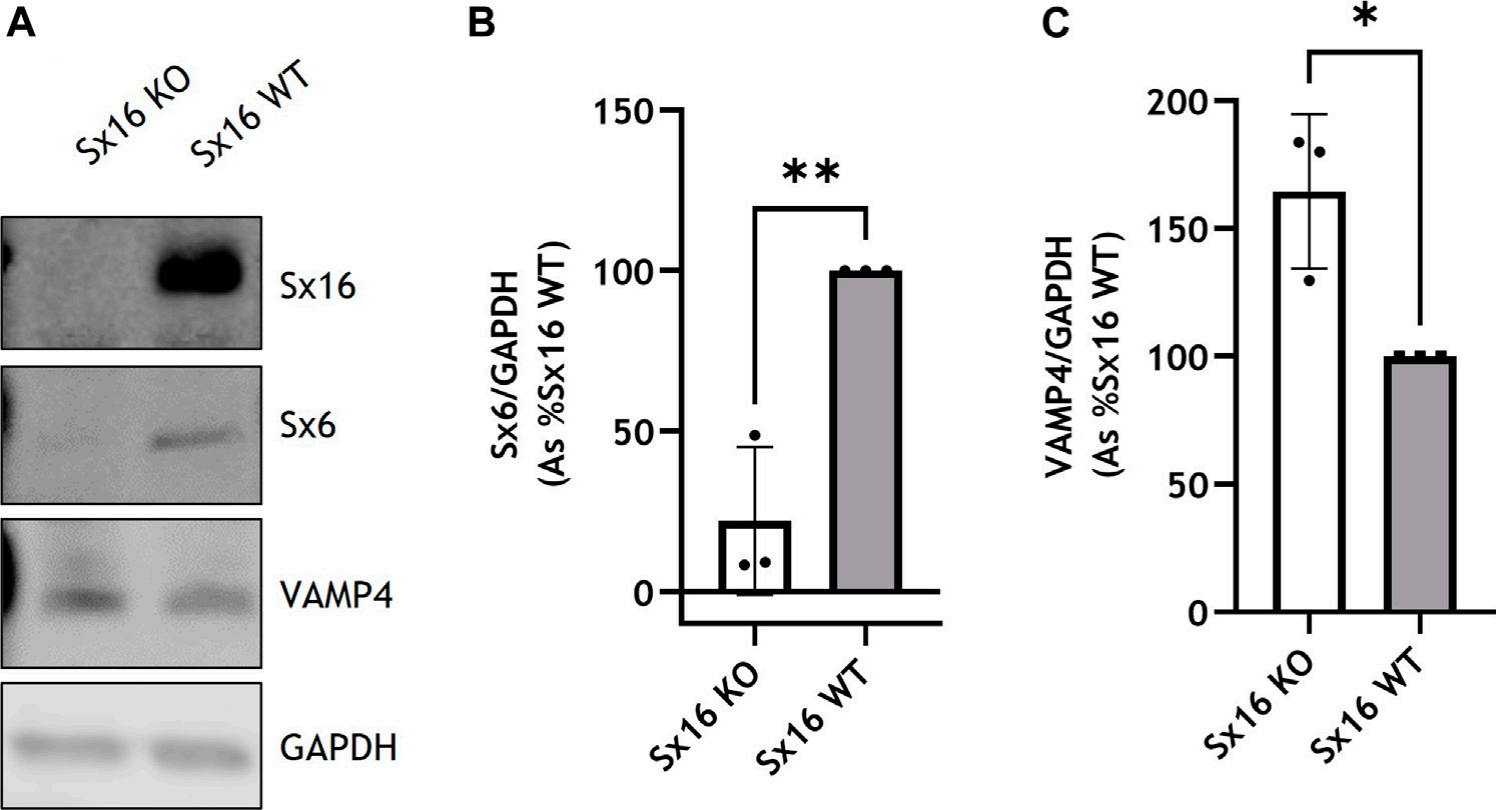
Generation of Sx16 knockout 3T3-L1 adipocytes. **(A)** Representative immunoblots of Sx16, Sx6, VAMP4 and GAPDH in Sx16 knockout (Sx16 KO) and wild-type (Sx16 WT) cells from a typical experiment is shown. Quantification of Sx6 **(B)** or VAMP4 **(C)** were determined by comparison with GAPDH using densitometric analysis. Data shown represent the mean ± SD of three independent experiments, each data point is shown. Data is presented as % relative to Sx16 wild type cell lysate. *p < 0.05 and **p < 0.01 by Student’s *t*-test.

The expression of Sx16 was knocked out in 3T3-L1 cells utilizing CRISPR-Cas9 technologies. Genome editing caused a 2 base pair deletion in codon 9 of the Sx16 gene resulting in a frameshift mutation and a premature stop codon in amino acid position 15. Immunoblotting of Sx16 knockout adipocyte lysates revealed complete disappearance of the immunoreactive band at ∼43 kDa (Figure 1A). Sx16 is a Q_a_-SNARE thought to act together with Sx6 providing the Q_bc_ SNARE domains to a functional complex (Proctor et al., 2006). Interestingly, levels of Sx6 were also markedly decreased, consistent with cognate regulation of this tSNARE pair (Wang et al., 2017). By contrast, the cognate R-SNARE VAMP4, which usually localizes at the *trans* Golgi network (Steegmaier et al., 1999), expression increased upon knockout of Sx16 (Figure 1).

**FIGURE 2:**
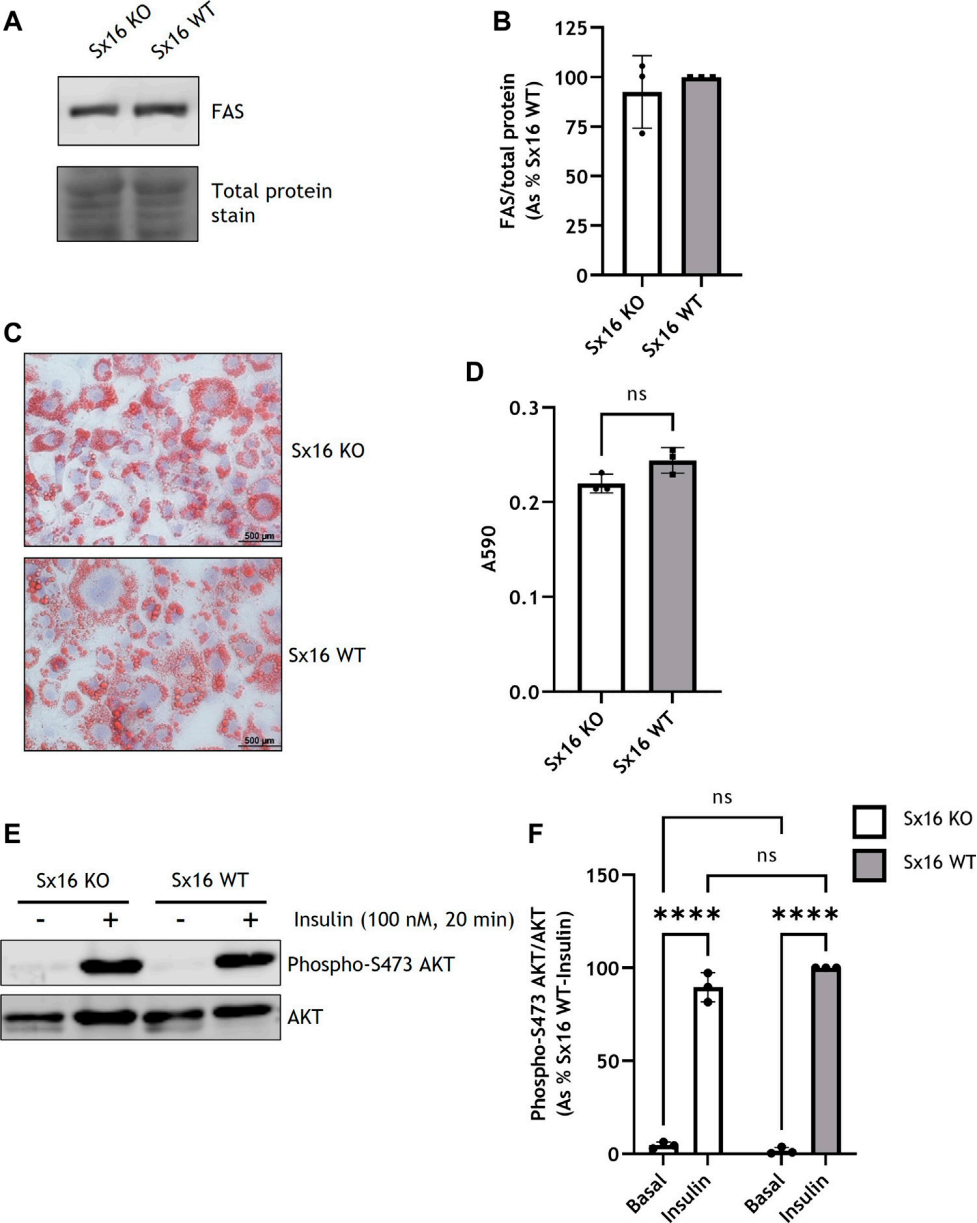
Sx16 knockdown does not affect 3T3-L1 adipogenesis. **(A)** Lysates prepared from Sx16 KO or WT cells were immunoblotted for fatty acid synthase (FAS) and stained for total protein with Revert™ total protein stain. **(B)** Quantification of FAS levels was determined by comparison with total protein using densitometric analysis. Data shown represent the mean ± SD of three independent experiments. Data is presented as % relative to Sx16 wild type cell lysate, **(C)** The indicated populations of adipocytes were stained with Oil Red O and Mayers Hematoxylin. Representative images are shown **(D)** Oil Red O stain was eluted from stained cells and absorbance at 590 nm measured. Data shown represent the mean ± SD of three independent experiments **(E)** Sx16-KO or WT cells were incubated with or without insulin as indicated and lysates prepared as described. Shown are representative immunoblots of phospho-S471 AKT and total AKT. **(F)** Quantification of AKT phosphorylation was determined by comparison with total AKT using densitometric analysis. Data is presented as % relative to insulin stimulated Sx16 wild type from three experiments of this type, each point on the graph is a single biological replicate. ****p < 0.0001 as assessed by two-way ANOVA.

To determine if Sx16 knockout and wild type cells differentiated to a similar extent, expression of the metabolic enzyme fatty acid synthase (FAS) was assessed and found to be at comparable levels in both cell lines (Figures 2A,B). Both genotypes differentiated to a similar extent and had comparable lipid content as assessed by Oil Red O staining (Figures 2C,D). Cells incubated with 100 nM insulin caused a comparable increase in AKT S473 phosphorylation, indicating that, at this concentration, insulin sensitivity was not altered in Sx16 knockout cells (Figures 2E,F). Together these data suggest that Sx16 has a role in regulating the expression of its cognate SNARE proteins Sx6 and VAMP4, but does not have an indispensable role in adipogenesis.

### 3.2 Sx16 knockout adipocytes have altered GLUT4 levels and trafficking

**FIGURE 3:**
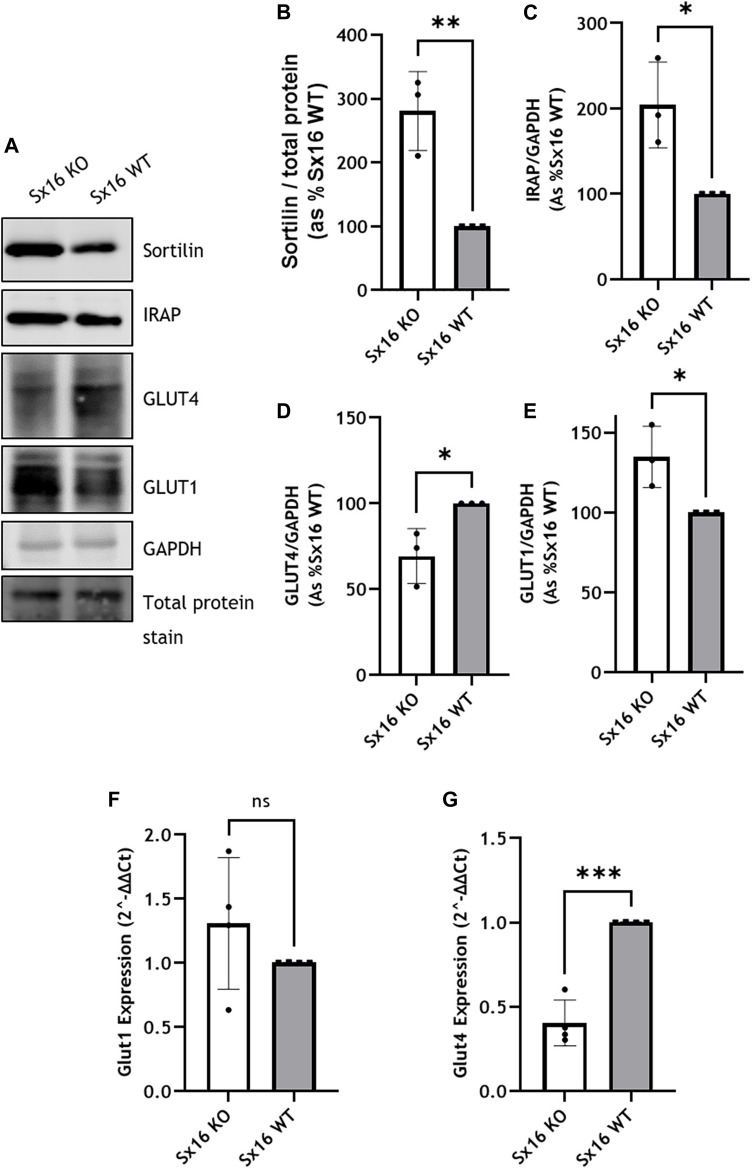
Glucose transporter expression in Sx16 knockout adipocytes. **(A)** Lysates prepared from Sx6 KO or WT cells were immunoblotted for the indicated proteins. Representative immunoblots of sortilin, insulin-regulated aminopeptidase (IRAP), glucose transporter 4 (GLUT4), GLUT1 and GAPDH are shown **(B–E)**. Quantification of Sortilin **(B)**, IRAP **(C)**, GLUT4 **(D)** or GLUT1 **(E)** were determined by comparison with GAPDH using densitometric analysis; data is from three biological replicates (mean ± SD, and each data point is shown **(F,G)** GLUT1 and GLUT4 gene expression was quantified using qPCR as outlined. Shown is the mean ± SD of four independent experiments in which the levels of GLUT4 and GLUT1 mRNA were quantified compared to hypoxanthine-guanine phosphoribosyl-transferase. Data is presented as % relative to Sx16 wild type cell lysate. *p < 0.05, **p < 0.01 and ***p < 0.001 by Students *t*-test.

In 3T3-L1 adipocytes, glucose transport is mediated by GLUT1 and GLUT4. Sx16 knockout adipocytes were found to have a 30 ± 9% reduction in GLUT4 levels compared to wild type cells (Figures 3A,D). This reduction is similar to that reported using transient Sx16 knockdown approaches (Proctor et al., 2006). Examination of sortilin and IRAP, key machinery required for GLUT4 sorting, indicated that both proteins were upregulated by 181 ± 36% (*p* < 0.01) and 104 ± 29% (*p* < 0.05) respectively (Figures 3A–C)). This may represent an adaptive response to declining GLUT4 levels, a point discussed further below. Analyzing mRNA levels in these adipocytes indicated a significant reduction (*p* = 0.001) in GLUT4 expression in Sx16 knockout cells whereas GLUT1 mRNA level was not significantly altered (Figure 3 F, G).

**FIGURE 4:**
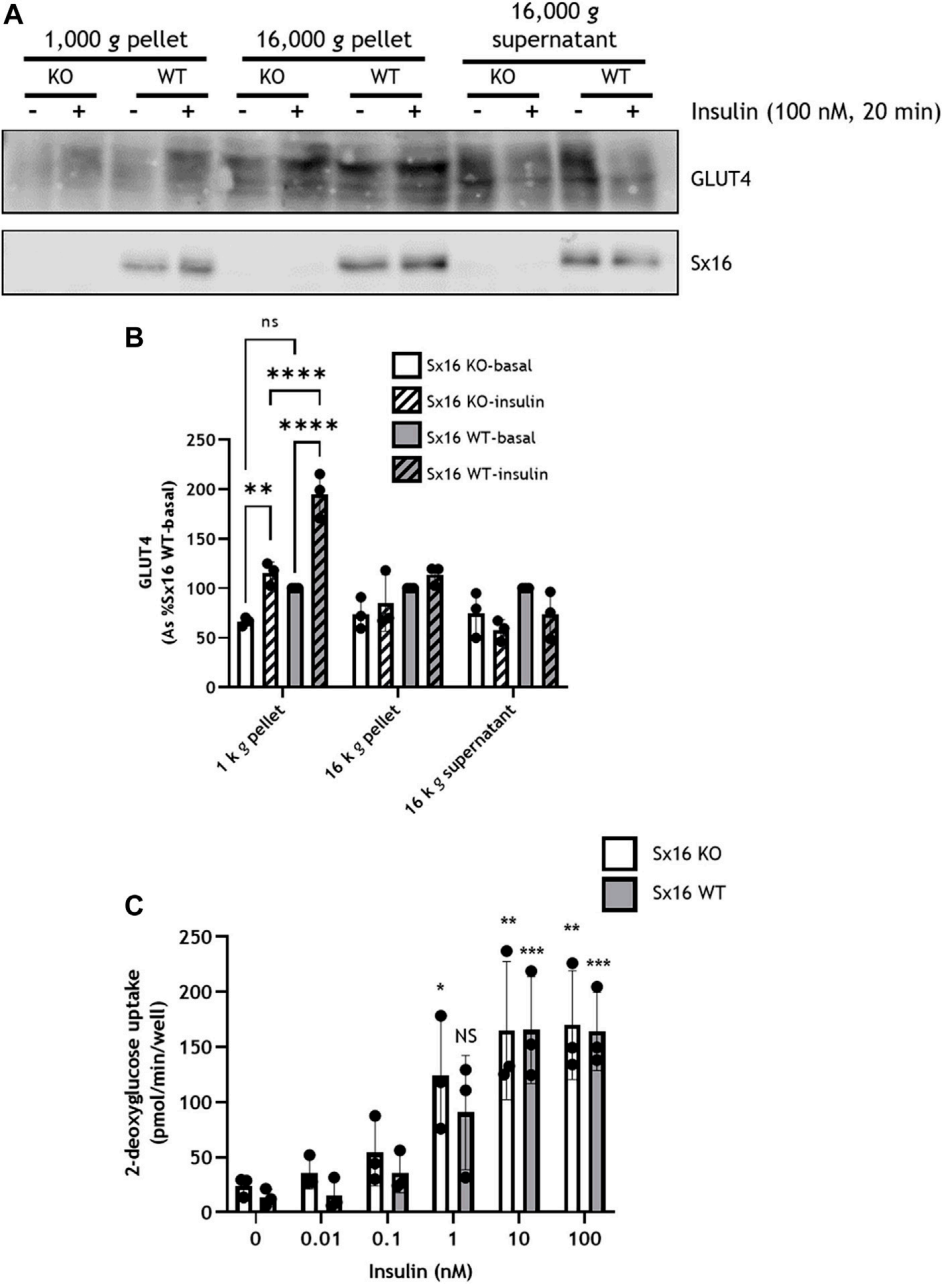
Insulin stimulated GLUT4 translocation but not glucose uptake was reduced in Sx16 knockdown cells. **(A)** Cell homogenates from Sx16-KO or WT cells were subjected sequential centrifugation to generate the 1,000 *g* pellet, 16,000 *g* pellet and the 16,000 *g* supernatant. The 1,000 g fraction is enriched for the PM and the 16,000 *g* supernatant is enriched in GSC/IRV (see text and Supplementary Figure S1). Representative immunoblots for GLUT4 and Sx16 are shown **(B)** Quantification of GLUT4 by densitometric analysis. Data is presented as % basal Sx16 wild type for each fraction from three independent experiments shown as mean ± SD. Significant effects of insulin on GLUT4 levels in the 1,000 *g* pellet are shown, together with significant differences between Sx16-KO and WT cells (**p < 0.01 and ****p < 0.0001 by two-way ANOVA; n.s. = not significantly different) **(C)** Glucose uptake was measured by the accumulation of 2-[^3^H] deoxyglucose in cells treated with or without the indicated concentrations of insulin for 30 min as outlined. Each data point on panel C are the means of three biological replicates ±SD (each from triplicate technical replicates). No significant difference was observed between Sx16 knockout and Sx16 wild type cells under any condition. Significant effects of insulin are indicated by *p < 0.05, **p < 0.01, ***p < 0.001; n.s. = not significantly different.

To examine GLUT4 translocation, adipocytes were homogenized and subjected to subcellular fractionation by differential centrifugation; the 1,000 *g* pellet is enriched for the plasma membrane (PM), whereas the 16,000 *g* pellet contains high density membranes (HDM) including the Golgi apparatus and endoplasmic reticulum (Gould et al., 1989; Black et al., 2022)—see also Supplementary Figure S1 which reveals that Sx4, a PM-localised tSNARE is enriched in the 1,000 *g* pellet (Olson at al., 1997). The resulting 16,000 *g* supernatant contained the cytosol and low density plasma membranes including IRVs (Sadler et al., 2016) and is enriched for VAMP4, a marker of the TGN (Supplementary Figure S1) (Tellam et al., 1997). The PM-enriched fraction of wild type exhibited a 95 ± 23% increase in GLUT4 levels in response to insulin. However, in Sx16 knockout cells insulin stimulated increases in GLUT4 in this fraction were reduced to 49 ± 7% increase in GLUT4 (Figures 4A,B). The 1,000 *g* pellet of insulin stimulated wild type cells also contained significantly (*p* < 0.0001) more GLUT4 than Sx16 knockout cells (Figure 4B). A similar trend was evident in unstimulated cells although this did not achieve statistical significance (*p* = 0.068).

Despite altered GLUT4 translocation, neither basal glucose transport, maximally insulin-stimulated glucose transport or the insulin sensitivity of glucose transport at multiple submaximal insulin concentrations were affected by Sx16 knockout (Figure 4C). This is distinct from data observed in transient knockdown experiments in which a modest reduction of insulin-stimulated glucose transport was reported (Proctor et al., 2006). This may represent an adaptive response to prolonged absence of Sx16, and consistent with this, we observed that Sx16 knockout cells exhibit elevated levels of GLUT1 protein (a 35 ± 11% increase in Sx16 knockout cells (Figure 3).

Collectively these data support previous studies indicating a role for Sx16 in GLUT4 trafficking, and substantiate that knockdown of Sx16 is accompanied by reduced GLUT4 levels, impaired translocation and a number of adaptive responses, including up-regulation of proteins involved in GSC biogenesis and increased expression of GLUT1 protein.

### 3.3 Adaption to hyperinsulinemia in Sx16 knockout adipocytes is altered

**FIGURE 5:**
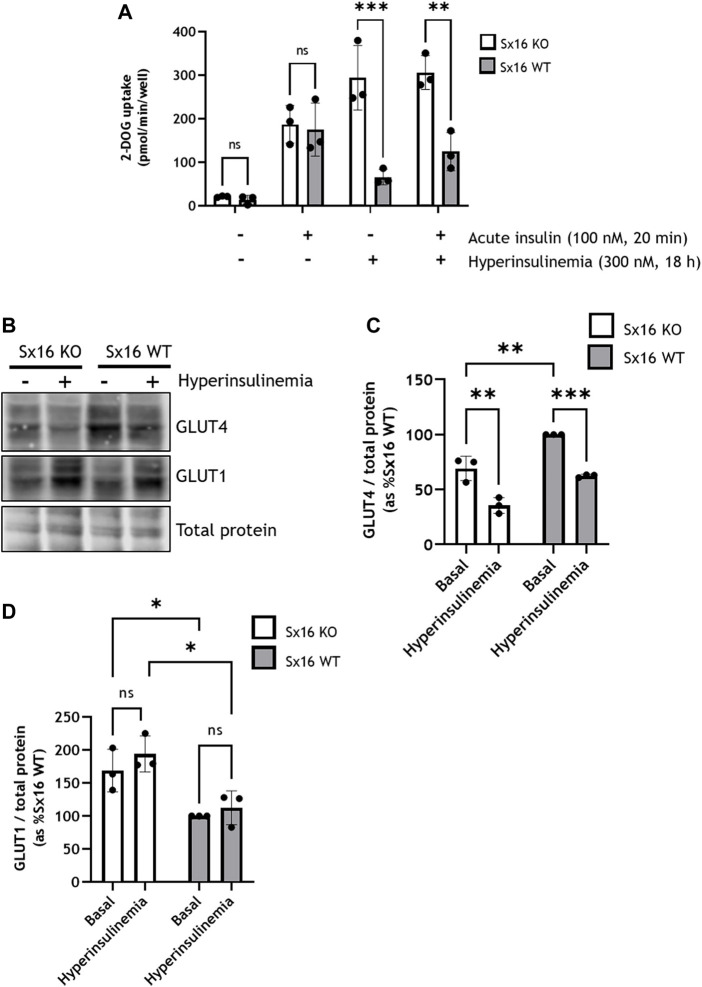
Sx16 knockout adipocytes have an altered response to hyperinsulinemia. 3T3-L1 adipocytes were incubated with 300 nM insulin for 18 h to induce insulin resistance (‘hyperinsuinemia’) of left untreated (control) **(A)** Glucose uptake was measured by the accumulation of 2-[^3^H] deoxyglucose. In these experiments cells were incubated without insulin or acutely challenged with insulin, as shown, prior to assay of deoxyglucose uptake. Each data point on panel C are the means of three biological replicates **(B)** Lysates from cells under these conditions were prepared and immunoblotted for GLUT4 and GLUT1 **(C–D)** Quantification of GLUT4 **(C)** or GLUT1 **(D)** was determined by comparison with total protein using densitometric analysis. Data shown represent the mean ± SD of three independent experiments. *p < 0.05, **p < 0.01, ***p < 0.001 by two-way ANOVA; n.s. = not significant.

The development of T2DM is often preceded by hyperinsulinemia. Given the important role of adipocytes in regulating metabolic status (Abel et al., 2001; B. B. Kahn, 2019; Santoro et al., 2021), we examined the effect of chronic incubation of 3T3-L1 adipocytes with insulin to ascertain how impaired GLUT4 trafficking may impact cellular physiology (Kozka & Holman, 1993; Maier & Gould, 2000). Consistent with published studies (Kozka & Holman, 1993; Maier & Gould, 2000), chronic insulin treatment of wild type cells resulted in elevated basal rates of glucose transport and a reduction in insulin-stimulated glucose transport (Figure 5A). Strikingly, Sx16 knockout cells had a 15-fold increase in basal glucose uptake following hyperinsulinemia (Figure 5A). Next, the glucose transporter profile of these cells was examined; in both genotypes GLUT4 was significantly (*p* = 0.0011 for Sx16 knockout and *p* = 0.0006 for Sx16 wild type cells) downregulated in response to hyperinsulinemia whereas GLUT1 levels did not significantly change (Figures 5B–D).

**FIGURE 6:**
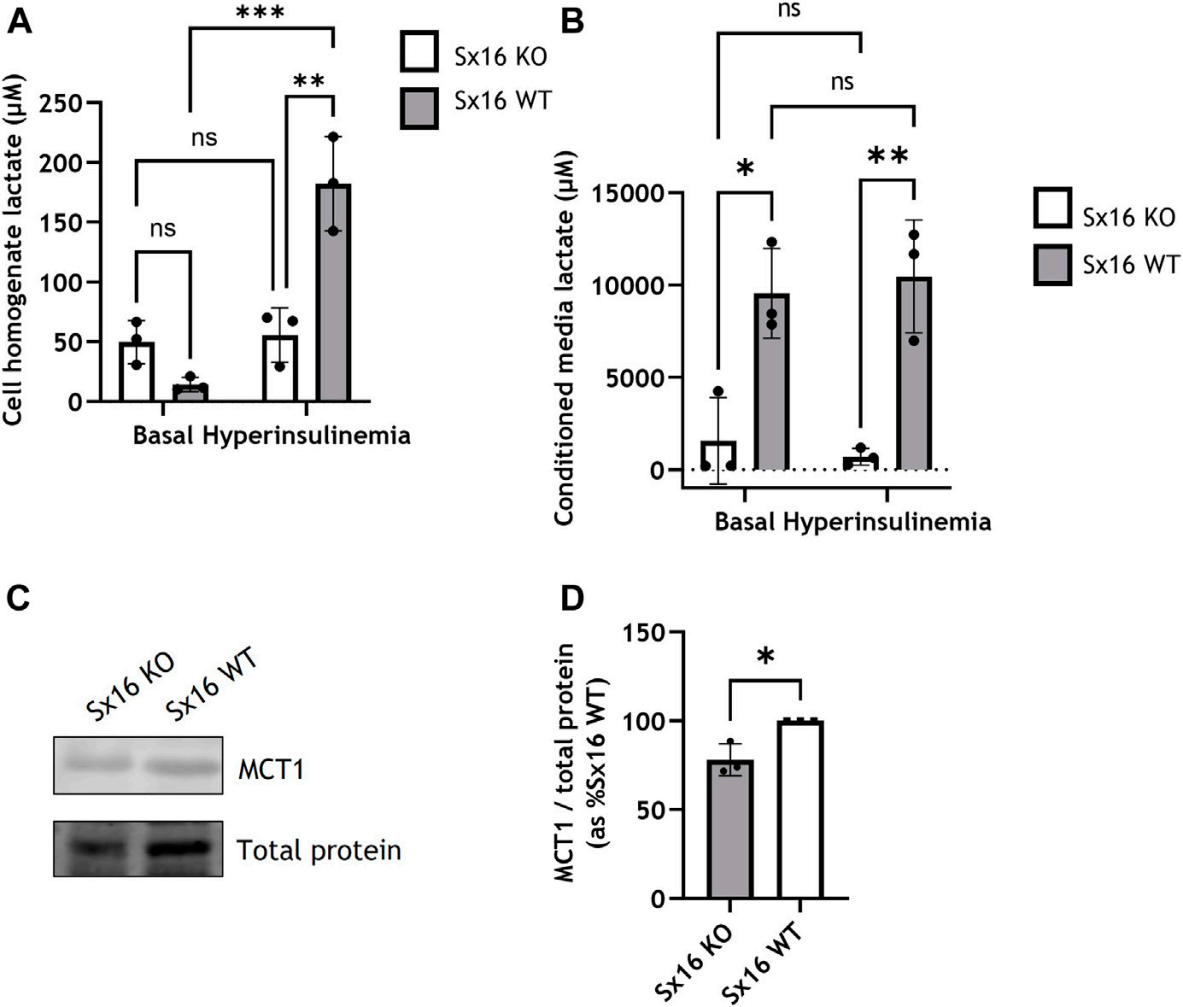
Sx16 knockout cells have altered lactate metabolism. Hyperinsulinemia was achieved by incubating cells with 300 nM insulin for 18 h in serum free media. Lactate concentration was determined from cell homogenates **(A)** or conditioned media **(B)**. **(C)** Cell lysates were immunoblotted for MCT1 and compared with total protein stain **(D)** Quantification of MCT1 was determined by comparison to the total protein stain using densitometric analysis from three biological replicates of each condition. Data shown represent the mean ± SD of three independent experiments. *p < 0.05, **p < 0.01, ***p < 0.001 by two-way ANOVA or Students *t*-test; n.s. = not significant.

To examine the metabolic changes which occurred in response to hyperinsulinemia, lactate content was measured. Unstimulated (basal) intracellular lactate was not significantly different between Sx16 wild type and knockout adipocytes (Figure 6A). However, following hyperinsulinemia Sx16 wild type exhibited a ∼12-fold increase in intracellular lactate (Figure 6A). Hyperinsulinemia did not influence intracellular lactate from Sx16 knockout cells. The conditioned media of Sx16 knockdown cells was found to contain significantly less lactate that wild type cells, indicating reduced lactate secretion (Figure 6B). Lactate secretion was unaltered in response to hyperinsulinemia in both cell lines (Figure 6B). The lactate transporter, MCT1 was found to be downregulated in Sx16 knockout cells (Figures 6C,D). Together, this indicates that Sx16 has a role in adipocyte adaptation to hyperinsulinemia and maintaining lactate flux.

### 3.4 Sx16 has a role in adipokine secretion

**FIGURE 7:**
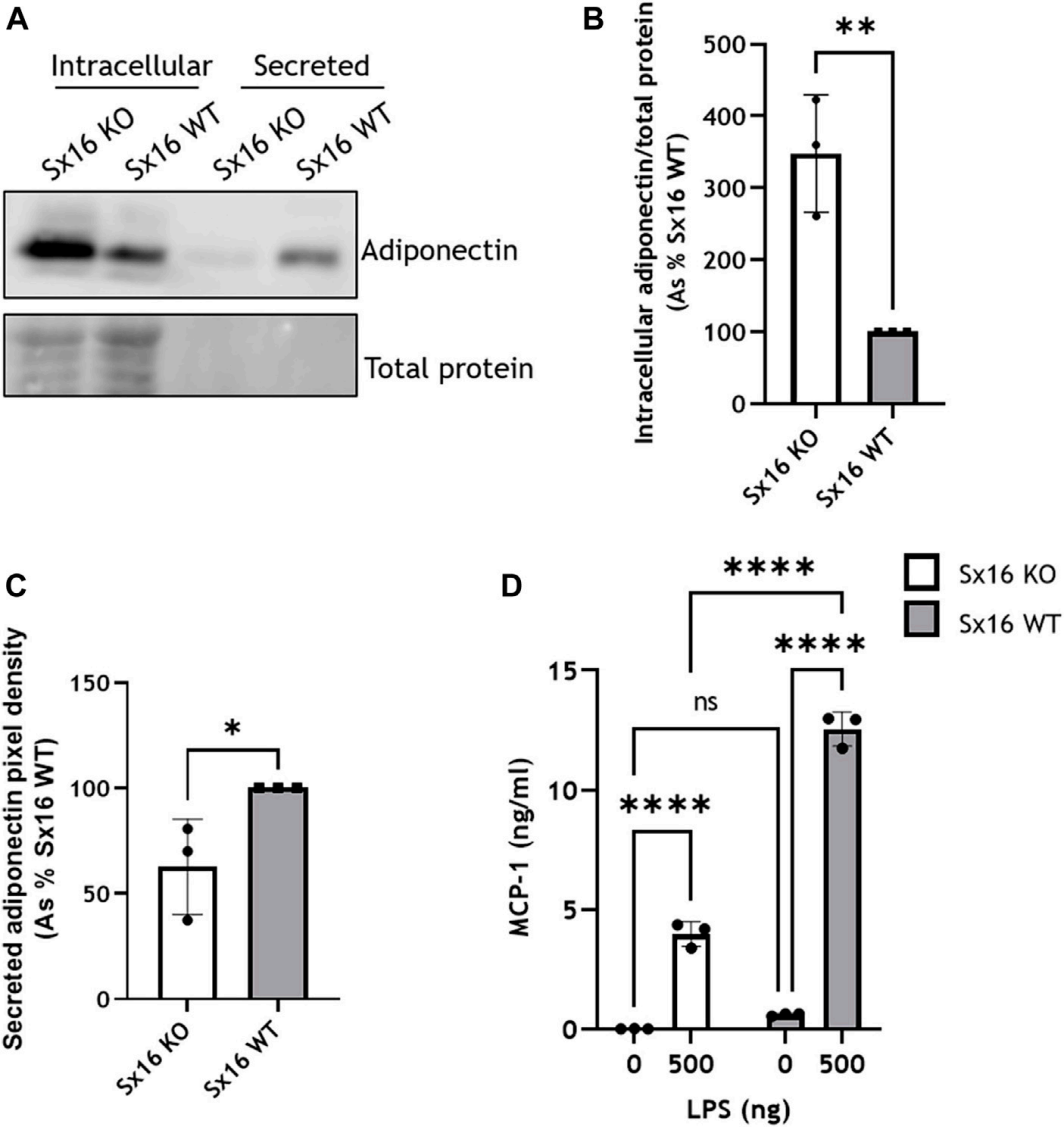
Adipokine secretion in impaired in Sx16 knockout adipocytes. **(A)** Adipocytes were cultured in serum free DMEM for 18 h prior to media collection and preparation of cell lysates from Sx16-KO or WT cells as described. Shown is a representative immunoblot of adiponectin and total protein stain **(B)** Quantification of intracellular adiponectin relative to total protein stain by densitometric analysis **(C)** Quantification of secreted adiponectin by densitometric analysis **(D)** Adipocytes were stimulated with 500 ng lipopolysaccharide (LPS) for 18 h in serum free media, conditioned media was collected and MCP-1 content assessed by Quantikine^®^ ELISA. Data shown represent the mean ± SD of three independent experiments. *p < 0.05, **p < 0.01, ***p < 0.001 and ****p < 0.0001; n.s. = non significant.

Adipocytes secrete a range of adipocytokines which play a key role in whole body energy metabolism (C. R. Kahn et al., 2019). The secretion of these proteins exhibits constitutive and regulated properties (Rajan et al., 2017). For example Adipsin release is acutely increased by insulin (Kitagawa et al., 1989). The secretion of these adipocytokines likely involves the *trans*-Golgi network (TGN) (Clarke et al., 2006). We therefore examined secretion of adipokines in Sx16 knockout cells. Adiponectin was found at higher levels intracellularly in Sx16 knockout cells. Conversely, Sx16 knockout adipocytes secreted significantly less (*p* < 0.001) adiponectin than wild type cells. (Figures 7A–C). Together this suggests that without Sx16, adiponectin accumulates intracellularly rather than being secreted.

The secretion of MCP-1 was examined following stimulation with LPS (Cullberg et al., 2014). LPS-induced a substantial increase of MCP-1 secretion in both Sx16 knockout and wild type cells but at considerably different magnitudes; the extracellular media from S×16 KO adipocytes contained 3.9 ± 0.5 ng/ml MCP-1, whereas wild type cells higher levels 11.7 ± 1.3 ng/ml (Figure 7D). Together, this indicates that Sx16 knockout adipocytes have defective adipokine secretion.

## 4 Discussion

Sx16 knockdown experiments have suggested a role for Sx16 in GLUT4 sorting (Proctor et al., 2006; Roccisana et al., 2013). However, the variability inherent in knockdown approaches, together with difficulties in transfecting adipocytes (see *limitations* below), have hampered wider understanding of the role of this important TGN Q_a_-SNARE. To address this, Sx16 knockout 3T3-L1 cells were generated by genome editing; in parallel, control lines were generated which were taken through the same clonal expansion processes to insure comparability of control cells and knockout lines. Sx16 knockout cells had a complete knockout of Sx16 protein expression. Sx16 has been reported to be upregulated in 3T3-L1 adipogenesis but a role for Sx16 in differentiation was not examined (Roccisana et al., 2013). Here we show that Sx16 expression does not affect the expression of the lipogenic protein FAS nor does it influence lipid accumulation. The cells also retain insulin-sensitivity, as assessed by AKT S473 phosphorylation and glucose uptake. These data suggest that Sx16 is not required for adipogenesis in this model.

Sx16 interacts with both Sx6 and VAMP4 (Mallard et al., 2002; Perera et al., 2003). Here we report that knockout of Sx16 significantly downregulated levels of the cognate Q_bc_-SNARE Sx6 whereas the proposed R-SNARE VAMP4 was upregulated. The overexpression of VAMP4 may reflect upregulation of an alternative SNARE pathway as plasticity of SNARE proteins has been well documented (Zhao et al., 2009; Sadler et al., 2015). Transient knockdown of Sx16 in 3T3-L1 adipocytes by electroporation of Sx16 morpholine antisense oligonucleotides only modestly affected Sx6 expression (Proctor et al., 2006). The observed downregulation of Sx6 in this study may be an adaptive response due to the complete lack of Sx16 rather than the transient effects observed by Proctor et al.

Our data indicate that Sx16 is not essential for GLUT4 trafficking. Insulin-stimulated GLUT4 translocation to the PM occurred in Sx16 knockout cells, albeit at reduced efficiency. Future experiments in which Sx16 is knocked out in cells expressing HA-GLUT4-GFP will allow a more accurate measure of GLUT4 translocation and recycling kinetics. It is important to note that multiple mechanisms to deliver GLUT4 into IRVs have been proposed (Bogan, 2012; Klip et al., 2019; Gould et al., 2020). These include a Sx16-dependent step from the TGN, and a Golgi by-pass route utilizing a novel clathrin coat, CHC22 in human cells (Camus et al., 2020). Protein complexes based on IRAP and sortilin have both been implicated, perhaps reflecting subtly different routes towards IRVs or each operating on a different step of the sorting pathway from TGN to GSV to IRV (Bogan, 2012; Klip et al., 2019; Gould et al., 2020). The upregulation of sortilin and IRAP we report here in response to Sx16 knockout may represent an attempt to compensate for the lack of Sx16, ensuring GLUT4 sorting into IRVs and translocation is maintained. Consistent with this, in Sx16 knockout cells, insulin-stimulated GLUT4 trafficking to the cell surface was reduced by approximately 50%, which may suggest that IRVs contained less GLUT4, the IRV population was reduced or that translocation occurred at a lower efficiency. We note that cellular GLUT4 levels were reduced in the knockout cells, perhaps reflecting changed intracellular itinerary for GLUT4.

Surprisingly neither basal- nor insulin-stimulated 2-deoxyglucose uptake was affected by Sx16 knockdown despite the altered expression profile of GLUT1 and GLUT4. It was expected that since GLUT4 expression was lowered and translocation concomitantly reduced, that insulin-stimulated transport would have been similarly lower in Sx16 knockout cells. It is unclear whether this is a reflection of only a modest change in GLUT4 levels or whether there are other mechanisms at play. Such mechanisms may include re-distribution of GLUT1, the expression of other GLUT isoforms (although we were unable to identify any changes in GLUT8 or GLUT10; Bremner unpublished), or alterations in intrinsic activity (which have been reported for GLUT1) (Harrison et al., 1991; Cloherty et al., 1996). We note that similarly modest reductions in GLUT4 levels were observed in other experimental systems and were accompanied by reduced rates of glucose transport (Proctor et al., 2006; Black et al., 2022). Although it has been documented that a 50% reduction of GLUT4 in murine skeletal muscles does not affect glucose uptake during exercise (Fueger et al., 2004).

It is of interest that GLUT1 levels were upregulated in our knockout line. GLUT1 exhibits insulin-stimulated delivery to the surface in 3T3-L1 adipocytes and has long been identified as a contributor to net glucose uptake in these cells. Thus, changes in levels of GLUT1 may in part compensate for reduced GLUT4 levels. It should also be noted that GLUT1 activity can also be modified, e.g., through oligomerisation (Hebert & Carruthers, 1992), or by targeting to lipid rafts (Kumar et al., 2004). Furthermore, protein-protein interactions have also been demonstrated to influence GLUT4 intrinsic activity. Both GAPDH and hexokinase-II are suggested to interact with GLUT4 to regulate its activity in response to insulin (Zaid et al., 2009). Further work is required to explain why reduced GLUT4 levels do not result in decreased glucose transport in these cells.

Downregulation of adipocyte GLUT4 in people with T2DM leads to systemic insulin resistance (Abel et al., 2001; W. T. Garvey et al., 1988; B. B. Kahn, 2018; Vargas et al., 2022). This is mimicked in adipose-specific GLUT4 knockout mice (Stenbit et al., 1997). In contrast, the adipose-specific overexpression of GLUT4 lowers fasting blood glucose levels and enhances glucose tolerance (Carvalho et al., 2005). Studies in human adipocytes and muscle point to defective GLUT4 sorting as a key defect in T2DM (Abel et al., 2001; Kahn, 2019; Santoro et al., 2021). Understanding the relative balance of trafficking pathways is important to gain a clear understanding of T2DM. Our data argue that loss of one of the major proteins involved in GLUT4 sorting, Sx16, can be at least partially buffered to maintain cellular GLUT4 at sufficient levels to exhibit insulin-stimulated glucose transport.

Given the central role for adipocyte GLUT4 in insulin sensitivity, we wondered whether the reduction of GLUT4 observed in our Sx16 knockout cells could have more significant implications in the context of disease aetiology. To test this, we incubated 3T3-L1 adipocytes in high insulin concentrations as a model of hyperinsulinemia. In Sx16 wild type cells, this results in a characteristic increase in basal glucose transport, reduced insulin-sensitivity, and a modest reduction in GLUT4 levels likely arising from mis-targeting. In Sx16 knockout cells, hyperinsulinemia resulted in a dramatic elevation in basal glucose uptake, and a complete loss of insulin-stimulated transport. Hence, these data argue that Sx16 loss (or reduction) could have significant effects on whole body insulin sensitivity. Consistent with this, a reduction in Sx16 levels was observed in skeletal muscle of obese patients with diabetes compared to weight-matched controls (R. Livingstone and G.W. Gould, unpublished).

Disturbances in glycolytic metabolism are likely to have implications on whole cell metabolism. The rate of cellular glucose transport can be regulated by mechanisms other than the glucose transports, such as intracellular glucose metabolism. This was exemplified by a study in which glycolytic flux was measured in cells overexpressing individual components of each step of the glycolytic pathway from GLUT1 to lactate transporters. This study revealed that increases at any of these steps, including hexokinase and phosphofructokinase, could drive increased glycolytic flux (Tanner et al., 2018). This prompted us to consider that adipocytes with altered GLUT levels may also exhibit alterations in intracellular glucose metabolism. Figure 6 indicates this is the case, at least as evidenced by changes in lactate levels. Levels of lactate released into the media are substantially lower in Sx16 knockout cells, however, intracellular lactate concentration is not significantly altered. Lactate secretion was unaffected by hyperinsulinemia in knockout adipocytes, but wild type cells had a ∼12-fold increase in cellular lactate. It should be emphasized that such differences are far more readily identified in knockout cell lines than is the case in heterogeneous knockdowns. The basis of this effect is not clear but could suggest that alterations in trafficking and/or functional activity of members of the lactate transporters, such as MCT1, are regulated in a Sx16-dependent manner as MCT1 expression was subtly but significantly downregulated. Other possible explanations include perturbation of oxidative phosphorylation, pentose phosphate metabolism or lactate synthesis. Further work will be required to clarify this important point.

Adipocytes secrete a range of adipocytokines which act as both paracrine and autocrine regulators of cell function. Here we show that knockout of Sx16 significantly impairs secretion of two adipocytokines; adiponectin and MCP-1. Constitutive adiponectin secretion is significantly impaired in Sx16 knockout cells, with an associated increase in intracellular adiponectin levels. This is consistent with the hypothesis of impaired TGN sorting in Sx16 knockout cells. Interestingly, regulated secretion is also impaired in Sx16 knockout cells, where we observed decreased LPS-stimulated MCP-1 secretion.

Limitations of the study. Knockout of any gene using CRISPR technology is open to the criticism of off-target effects. These are often addressed by ‘add-back’ experiments in which the originally deleted gene is restored. We have been unable to achieve this in 3T3-L1 adipocytes. Stable Sx16 knockout cells selected to express ectopic Sx16 failed to exhibit any differentiation into adipocytes. This is a common issue with this cell line, whose capacity to differentiate into adipocytes is finite and limits such analyses. Similarly, these cells are notoriously difficult to transfect, preventing even simple DNA-mediated gene transfer with any degree of consistency of efficiency, an issue which also limits ability to measure GLUT4 translocation using HA-GLUT4 or GLUT4-myc, as these cannot be introduced into cells with sufficient efficiency. Hence, we acknowledge that our study has limits, based on this point. We would however note that the results obtained using Sx16 knockout are broadly in agreement with studies using either knockdown with morpholino anti-sense oligonucleotides or expressing mutant forms of Sx16 which act as dominant negative inhibitors. The use of multiple gRNAs would be a useful addition to future work of this type. The important addition offered by this system is a robust cell line that can be readily propagated and expanded to allow more detailed biochemical analysis than can readily and affordably achieved using siRNA.

Although we have observed metabolic perturbations in Sx16 knockout cells, clearly further and detailed metabolomic studies would be required to establish the mechanistic basis of this. It is of interest to note that recent work from our laboratory examining obese patients with and without Type-2 diabetes revealed that diabetes is accompanied by a reduction in Sx16 levels in skeletal muscle (Livingstone et al., 2022). Such data provide further support for the idea that Sx16 is important for GLUT4 trafficking.

In summary, we show that Sx16 is involved in maintaining GLUT4 expression. In the absence of Sx16, adipocytes still exhibit robust insulin-stimulated glucose transport but exhibit altered behavior in response to hyperinsulinemic conditions and changed metabolic profiles, a result with potentially important implications for the development of metabolic disease. We further show that Sx16 plays a role in both regulated and constitutive secretion of adipokines. Our data provide further support for the hypothesis that adipocyte GLUT4 levels are crucial for the control of systemic insulin resistance and suggest this may arise via effects both on adipocyte metabolism and on secretion of key adipokines.

## Data Availability

The raw data supporting the conclusions of this article will be made available by the authors, without undue reservation.

## Supplementary material

Supplementary Figure S1 The cell fractions corresponding to those shown in Figure 4A from wild-type cells treated with or without insulin were probed for Sx4 and VAMP4 as markers for the plasma membrane and light microsomes, respectively. These data reveal that Sx4, a PM-associated SNARE protein, is enriched in the 1,000 *g* fraction. VAMP4 is localized predominantly to the 16,000 *g* supernatant fraction, together with Sx16 and GLUT4 (see Figure 4).
