# Supplementary figures and images for: Pleiotropic effects of Syntaxin16 identified by gene editing in cultured adipocytes
Source: Front Cell Dev Biol. 2022 Nov 18;10:1033501. doi: 10.3389/fcell.2022.1033501 (PMC9716095; doi:10.3389/fcell.2022.1033501)

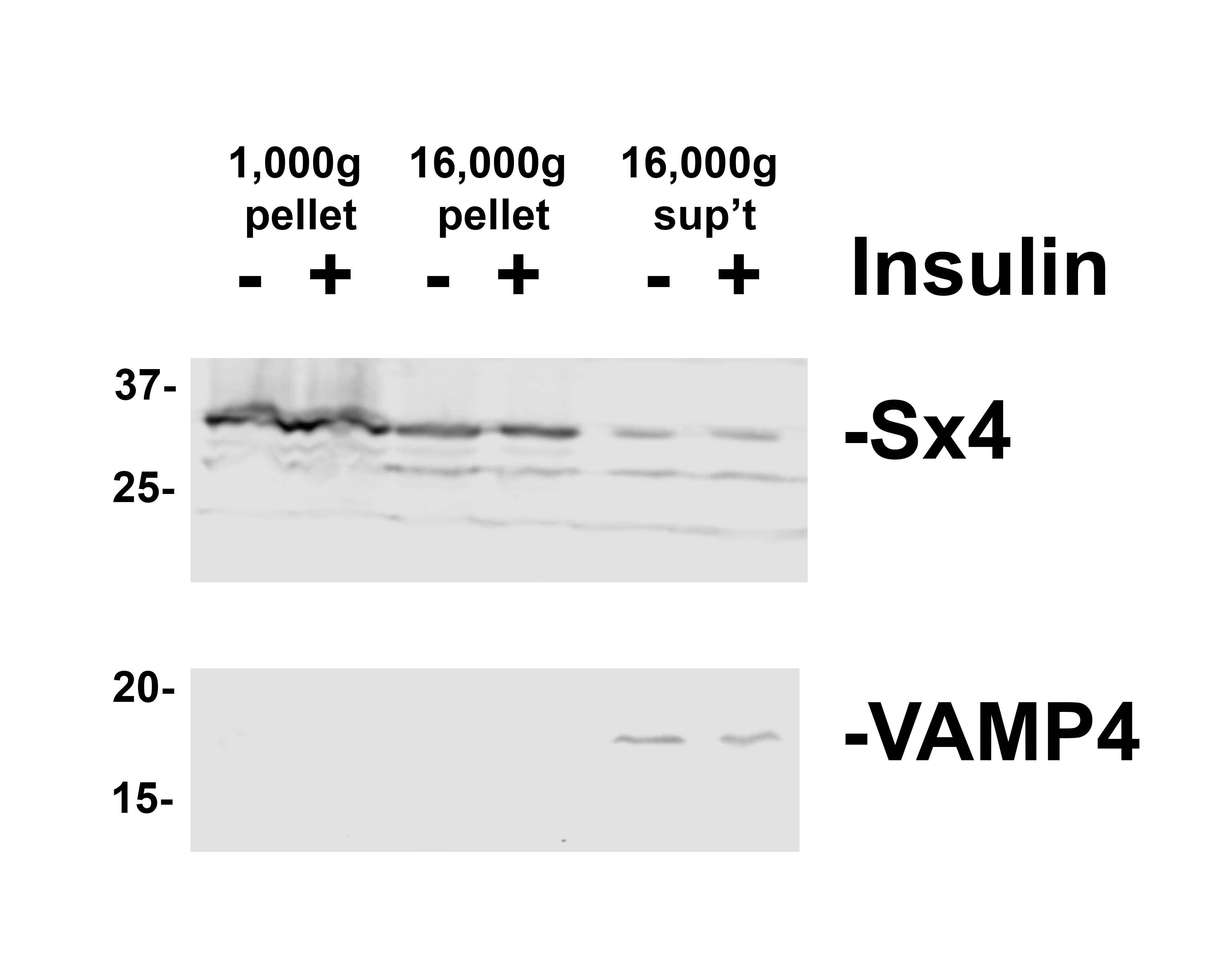

Supplement: Supplementary file 1 [file Image1.jpg]
